# Overwinter Syndrome in Grass Carp (*Ctenopharyngodon idellus*) Links Enteric Viral Proliferation to Mucosal Disruption via Multiomics Investigation

**DOI:** 10.3390/cells15020157

**Published:** 2026-01-15

**Authors:** Yang Feng, Yi Geng, Senyue Liu, Xiaoli Huang, Chengyan Mou, Han Zhao, Jian Zhou, Qiang Li, Yongqiang Deng

**Affiliations:** 1 Fisheries Research Institute, Sichuan Academy of Agricultural Sciences, Chengdu 611731, China; fyang@scsaas.cn (Y.F.); liusenyue@scsaas.cn (S.L.); chengyanmou4@scsaas.cn (C.M.); zhaohan232323@163.com (H.Z.); zhoujian980@126.com (J.Z.); 2 Key Laboratory of Aquatic Health and Intelligent Aquaculture of Sichuan Province, Chengdu 611731, China; hxlscau@126.com; 3 College of Veterinary Medicine, Sichuan Agricultural University, Chengdu 611130, China; gengyisicau@126.com; 4 Department of Aquaculture, College of Animal Science & Technology, Sichuan Agricultural University, Chengdu 611130, China

**Keywords:** overwinter syndrome, grass carp, intestinal health, microbiota, host–microbe interaction

## Abstract

**Highlights:**

**What are the main findings?**

- OWS involves triple-barrier failure: physical, microbial, and immune disruption in grass carp intestine.
- Caudoviricetes phages expand and correlate with host gene dysregulation.

**What are the implications of the main findings?**

- Viral proliferation is linked to disrupted nucleotide metabolism and mucosal immunity.
- Gut microbiota shows elevated richness but stable diversity, reflecting viral-driven dysbiosis without a dominant bacterial pathogen.

**Abstract:**

Overwinter Syndrome (OWS) affects grass carp (*Ctenopharyngodon idellus*) aquaculture in China, causing high mortality and economic losses under low temperatures. Failure of antibiotic therapies shows limits of the ‘low–temperature–pathogen’ model and shifts focus to mucosal barrier dysfunction and host–microbiome interactions in OWS. We compared healthy and diseased grass carp collected from the same pond using histopathology, transcriptomics, proteomics, and metagenomics. This integrated approach was used to characterize intestinal structure, microbial composition, and host molecular responses at both taxonomic and functional levels. Results revealed a three-layer barrier failure in OWS fish: the physical barrier was compromised, with structural damage and reduced mucosal index; microbial dysbiosis featured increased richness without changes in diversity or evenness, and expansion of the virobiota, notably *uncultured Caudovirales phage*; and mucosal immune dysregulation indicated loss of local immune balance. Multi-omics integration identified downregulation of lysosome-related and glycosphingolipid biosynthesis pathways at transcript and protein levels, with disrupted nucleotide metabolism. Overall gut microbial richness, rather than individual taxa abundance, correlated most strongly with host gene changes linked to immunity, metabolism, and epithelial integrity. Although biological replicates were limited by natural outbreak sampling, matched high-depth multi-omics datasets provide exploratory insights into OWS-associated intestinal dysfunction. In summary, OWS entails a cold-triggered breakdown of intestinal barrier integrity and immune homeostasis. This breakdown is driven by a global restructuring of the gut microbiome, which is marked by increased richness, viral expansion, and functional shifts, ultimately resulting in altered host–microbe crosstalk. This ecological perspective informs future mechanistic and applied studies for disease prevention.

## 1. Introduction

Overwintering syndrome (OWS) is a seasonal disease of grass carp (*Ctenopharyngodon idellus*) in China that typically coincides with prolonged cold spells and causes severe welfare and production losses. In affected ponds, fish cease feeding and develop lethargy and skin ulcers, with reported mortality reaching 30–90% under intense farming conditions. OWS has long been linked to sustained low temperatures (water ≤ 12 °C for >5 days) that induce metabolic and immune suppression [1]. Cold stress in grass carp markedly elevates oxidative stress and inflammation [2], and immunosuppression further predisposes fish to secondary infections by opportunistic pathogens (e.g., *Flavobacterium psychrophilum* and *Aeromonas* spp.) [3]. However, routine antibiotic treatment of OWS is often ineffective, implying that the pathology is not solely due to cold or infection. Recent evidence from gill tissues has shifted the focus beyond singular pathogens, revealing that OWS is associated with a state of systemic dysbiosis and a “Dysbiosis–Ferroptosis–Collapse” axis, wherein microbiota-driven barrier disruption promotes non-resolving inflammation and tissue damage [4]. Beyond suppressing immune function, sustained low temperature and environmental stress are likely to cause severe damage to the mucosal barriers, especially the gut, thereby compounding immune failure.

As the primary site for nutrient uptake and immune defense, the intestinal mucosal barrier consists of a monolayer of epithelial cells linked by tight junctions, covered with a protective mucus layer, and inhabited by a dense commensal microbiota. This barrier normally prevents pathogen invasion and maintains homeostasis, but it is vulnerable to environmental insults. Indeed, studies have shown that stressors such as extreme cold and ammonia nitrogen can severely impair fish intestinal integrity: for example, cold shock causes shortening and atrophy of intestinal villi and disruption of tight junctions, while high ammonia leads to structural damage, fewer mucus-producing goblet cells, and degraded tight junctions [5,6]. We therefore hypothesize that during OWS, chronic cold stress depletes the mucus layer and damages epithelial cells in the gut, causing barrier breakdown.

A healthy gut microbiota normally supports the intestinal barrier and prevents pathogen overgrowth through nutrient competition, production of antimicrobial factors, and modulation of host immunity [7]. Conversely, impaired barrier function can trigger dysbiosis of the gut flora, creating a vicious cycle of deteriorating health [8,9]. In fish, barrier disruption allows bacteria and their toxins to translocate into the tissue and bloodstream, provoking intense local and systemic inflammation [10,11]. We thus anticipate that in OWS, cold-induced barrier failure and dysbiosis mutually reinforce each other, accelerating fish decline. To test this, we will characterize changes in the gut microbial community and the physical condition of the intestinal barrier during overwintering. By linking dysbiosis with measures of barrier damage and inflammation, our study aims to clarify the pathogenesis of OWS and inform disease-prevention strategies that prioritize ecological and mucosal health over antibiotic reliance.

## 2. Materials and Methods

### 2.1. Sample Collection and Necropsy

A sample of five grass carp exhibiting typical OWS signs (e.g., periocular and facial redness/swelling/ulceration, snout tip lesions, and abscesses on the back and caudal peduncle) was obtained from affected ponds in Zhongjiang County, Sichuan Province, China, in 2025. For comparison, four healthy fish were concurrently collected from the same location. The average body weight and total length were 544.98 ± 90.69 g and 36.36 ± 2.13 cm for the OWS-affected group, and 507.8 ± 88.14 g and 35.72 ± 1.84 cm for the healthy control group. All specimens were euthanized humanely using MS-222. A systematic post-mortem examination was then performed, which involved a detailed assessment of external and internal pathological changes and an analysis of alimentary tract contents. All omics analyses (histology, transcriptomics, proteomics and metagenomics) were performed on matched samples from the same individual fish. Specifically, each fish provided intestinal tissue for histology, RNA for transcriptomics, protein for proteomics, and intestinal content for metagenomic sequencing. Because samples were collected during a natural pond-based OWS outbreak, additional matched samples could not be obtained retrospectively under identical environmental conditions. The study complied with ethical guidelines for animal experiments from the Sichuan Academy of Agricultural Sciences, with approval from the Animal Care and Use Committee (permit No. 20250324001A).

### 2.2. Histopathological Analysis

Intestinal segments were collected from both healthy and diseased grass carp (*n* = 4 per group) and fixed in 10% neutral buffered formalin for tissue architecture preservation. The samples were then trimmed, processed through a graded ethanol series for dehydration, cleared with xylene, and embedded in paraffin wax. Subsequently, 4 μm sections were prepared using a rotary microtome (Leica RM2235, Leica Microsystems, Wetzlar, Germany). For histological evaluation, the sections were stained with hematoxylin and eosin (H&E) for general morphology or with Alcian Blue-Periodic Acid-Schiff (AB-PAS) staining (Servicebio, Wuhan, China) to identify mucous cells. All sections were examined and imaged using a light microscope (Olympus BX53, Olympus Corporation, Tokyo, Japan).

### 2.3. Transcriptomic Analysis

The transcriptomic analysis utilized intestinal tissues from three healthy and three diseased grass carp. Total RNA was extracted using an Animal RNA Extraction Kit (Majorbio, Shanghai, China). RNA concentration was measured with a NanoDrop2000 (Thermo Fisher Scientific, Wilmington, DE, USA), while integrity was confirmed by 1% agarose gel electrophoresis and an Agilent 5300 Bioanalyzer (Agilent Technologies, Santa Clara, CA, USA) for RQN assessment. Poly(A) + mRNA was then enriched using magnetic Oligo(dT) beads. First, the enriched mRNA was fragmented to 200–300 nt in divalent cation buffer at 94 °C. First-strand cDNA was synthesized from the fragmented RNA using the NEBNext^®^ Ultra™ II RNA Library Prep Kit (New England Biolabs, Ipswich, MA, USA). Second-strand cDNA synthesis was performed with dUTP incorporation to preserve strand orientation. The constructed libraries were adapter-ligated, size-selected to 300 ± 50 bp using AMPure XP Beads (Beckman Coulter, Brea, CA, USA), and amplified through 12 cycles of indexing PCR. Finally, the prepared libraries were sequenced on an Illumina NovaSeq 6000 platform (Illumina, San Diego, CA, USA).

For data analysis, the raw sequencing data first underwent adapter trimming and quality filtering using fastp, which removed short (<50 bp), low-quality (average quality < 20), and ambiguous reads. Following this, SortMeRNA was applied to filter out rRNA sequences by alignment to the SILVA SSU and LSU databases. The high-quality clean reads were then aligned to the grass carp reference genome (GCF_019924925.1) with HISAT2 (v2.1.0). Using the alignments, transcripts were reassembled with StringTie (v1.3.1) and their abundance was estimated by RSEM (v1.3.3) as TPM. Differential expression between groups was identified using DESeq2, with a Benjamini–Hochberg false discovery rate (*FDR*) < 0.05 and an absolute fold change ≥ 1.5 considered significant. Functional enrichment analysis was carried out based on the GO and KEGG databases using a hypergeometric test (*p* < 0.05). Furthermore, a protein–protein interaction network was reconstructed using STRINGdb (v11.5) with a high-confidence score threshold of 0.7.

### 2.4. Proteomic Analysis

Intestinal samples from grass carp (*n* = 3 per condition) were subjected to proteomic analysis. Total protein extraction was conducted under chilled conditions. First, tissues were homogenized on ice in a lysis buffer (8 M urea, 1% SDS) containing a protease inhibitor cocktail, using a high-throughput tissue grinder (3 cycles, 180 s each). This was followed by non-contact cryogenic sonication for 30 min. The homogenate was then centrifuged at 16,000× *g* for 30 min at 8 °C to collect the supernatant. Protein concentration was determined with a BCA assay kit (Thermo Scientific, Waltham, MA, USA), and quality was verified by SDS-PAGE.

For proteolytic digestion, 100 μg of protein was diluted in 100 mM TEAB buffer. The procedure involved reduction with 10 mM TCEP at 37 °C for 60 min and alkylation with 40 mM IAM at room temperature for 40 min in the dark. After centrifugation at 10,000× *g* for 20 min at 4 °C, the pellet was resuspended in TEAB and digested with trypsin (1:50, *w*/*w*) overnight at 37 °C. The resulting peptides were desalted using HLB columns, dried under vacuum, reconstituted in 0.1% TFA, and quantified with a NanoDrop One spectrophotometer (Thermo Scientific).

Data-independent acquisition (DIA) mass spectrometry was carried out on a timsTOF Ultra2 instrument (Bruker Daltonics, Bremen, Germany) coupled to a Vanquish Neo UHPLC system (Thermo Scientific). Peptide separation was performed on a uPAC capillary column (75 μm × 55 cm) using an 8-min gradient with solvents A (0.1% formic acid in water) and B (0.1% formic acid in 80% acetonitrile). The mass spectrometer was operated in DIA mode with positive ionization at 4.5 kV, collecting full-scan MS spectra from *m*/*z* 100 to 1700.

The raw DIA data were processed with Spectronaut (v19.0) by searching against a grass carp reference proteome. Key search parameters included: trypsin/P digestion with up to two missed cleavages, fixed carbamidomethylation (C), variable modifications of oxidation (M) and N-terminal acetylation, a peptide length range of 7–52, and an *FDR* threshold of 1% at both peptide and protein levels. Protein quantification was performed using Spectronaut default DIA-based quantification algorithms. Differential expression analysis was performed using a *t*-test in R, with significance defined as a fold change > 1.2 or <0.83 and a *p*-value < 0.05. Subsequent functional annotation involved GO and KEGG enrichment analyses, and protein–protein interaction networks were built using STRING (v11.5) with a confidence score > 0.7.

### 2.5. Metagenomic Sequencing

Intestinal content samples were aseptically collected from the lumen of healthy and diseased grass carp (*n* = 4 per group) using sterile instruments. These samples were immediately transferred to sterile tubes and stored at −80 °C until processing. Genomic DNA was extracted with the FastPure Stool DNA Isolation Kit (MJYH, Shanghai, China), quantified via a Quantus Fluorometer (Promega, Madison, WI, USA), and its integrity was confirmed by 1% agarose gel electrophoresis. The DNA was then fragmented to 350 bp using a Covaris M220 Focused-ultrasonicator (Covaris, Woburn, MA, USA). Sequencing libraries were prepared with the NEXTFLEX Rapid DNA-Seq Kit (Bioo Scientific, Austin, TX, USA) and subjected to sequencing on an Illumina NovaSeq™ X Plus platform (Illumina, San Diego, CA, USA).

For bioinformatic processing, adapter sequences and low-quality reads (length < 50 bp, average quality score < 20) were filtered out using fastp. Host–derived DNA was subsequently removed by alignment with BWA. The remaining high-quality reads were assembled into contigs using MEGAHIT (v1.2.9) under default parameters, retaining only those ≥300 bp. Open reading frames (ORFs) were predicted from these contigs with Prodigal, and genes shorter than 100 bp were excluded. Non-redundancy was achieved by clustering the gene set with CD-HIT (90% identity, 90% coverage), retaining the longest sequence from each cluster as the representative gene. Gene abundance was quantified as Counts Per Million (CPM) using SOAPaligner, and all relative abundances reported herein are expressed in CPM.

For functional and taxonomic annotation, the amino acid sequences of the non-redundant gene set were aligned against the NR database using Diamond (v2.0.15). Additional functional annotations were assigned using the eggNOG, KEGG, CARD, VFDB, and PHI databases. Beta-diversity was assessed based on the Bray–Curtis dissimilarity. Differential gene abundance in the diseased group was identified with the Wilcoxon rank-sum test. KEGG pathway enrichment was evaluated with corrections for multiple testing using the Bonferroni method. Furthermore, the LEfSe (Linear Discriminant Analysis Effect Size) method was employed, utilizing the non-parametric Kruskal–Wallis sum-rank test to identify gene groups exhibiting significant abundance differences between groups.

### 2.6. Statistical Analysis

Identification of differentially expressed genes was conducted with the DESeq2 (v1.30.1), utilizing raw count data. A significance threshold was set at an *FDR* of <0.05 in conjunction with an absolute fold change of ≥1.5. Proteomic differential expression analysis involved the application of Student’s *t*-test to log2-transformed protein intensity values, with proteins meeting the criteria of *p* < 0.05 and a fold change > 1.2 or <0.83 deemed significant. In metagenomic differential abundance analyses, non-parametric tests (Kruskal–Wallis or Wilcoxon rank-sum test) were applied as appropriate. Specifically, LEfSe analysis was conducted using the Kruskal–Wallis sum-rank test, and features with linear discriminant analysis (LDA) scores exceeding the predefined threshold were considered discriminatory. Furthermore, Spearman’s rank correlation coefficient was computed to assess relationships between microbial taxa and host molecules, and the resulting *p*-values were subjected to multiple testing correction by the Benjamini–Hochberg method. The R statistical environment (v4.0.3) served as the platform for all analyses unless specified. A post-hoc power assessment was furthermore conducted to evaluate the statistical sensitivity for detecting the large effect sizes observed for pivotal molecules and pathways.

## 3. Results

### 3.1. OWS Induces Systematic Pathological Alterations in Grass Carp

**Figure 1:**
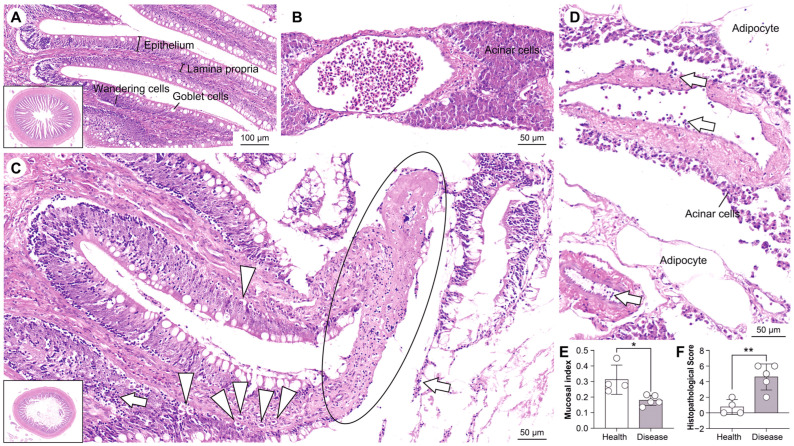
Pathological changes in the intestine of grass carp during OWS. (**A**): Histology of the intestinal mucosa in healthy grass carp. (**B**): Histology of the mesenteric pancreatic tissue in healthy grass carp. (**C**): Pathological changes in the intestinal mucosa during OWS; Circles indicate exposed and degenerated submucosal connective tissue, arrowheads indicate apoptotic cells, and arrows indicate infiltrating inflammatory cells. (**D**): Pathological changes in the mesenteric pancreatic tissue during OWS; Arrows indicate increased intravascular leukocytes. (**E**): Comparison of the mucosal index between groups (*n* = 4 per group); Mucosal index = mucosal thickness/intestinal diameter; Statistical significance was assessed using the Wilcoxon rank-sum test. (**F**): Comparison of histopathological scores between groups; Statistical significance was assessed using the Wilcoxon rank-sum test; Significance was indicated with asterisks, using the following conventions: *, *p* < 0.05; **, *p* < 0.01.

OWS induced systematic pathological alterations in grass carp that ranged from tissue morphology to molecular pathway perturbations, reflecting dysfunction along the intestine–pancreas axis. Histological analysis revealed that the intestinal mucosa of healthy grass carp displayed intact morphology and well-structured intestinal glands (Figure 1A), with no significant abnormalities in mesenteric pancreatic tissue (Figure 1B). In contrast, the OWS group displayed severe pathological damage in the intestinal mucosa, including overall shortening of intestinal villi, extensive exposure and necrotic degeneration of the submucosal connective tissue secondary to epithelial cell exfoliation, and prominent apoptosis with inflammatory cell infiltration (Figure 1C). Mesenteric pancreatic tissue also exhibited significant lesions, characterized by a marked increase in intravascular leukocytes and disorganized, reduced pancreatic acinar cells (Figure 1D). Quantitative comparisons showed that the mucosal index was significantly lower in the OWS group (Figure 1E), while the histopathological score was significantly higher, indicating moderate-to-severe pathological changes (Figure 1F).

### 3.2. Multi-Omics Analyses Reveal Transcriptional and Translational Dysregulation During OWS

**Figure 2:**
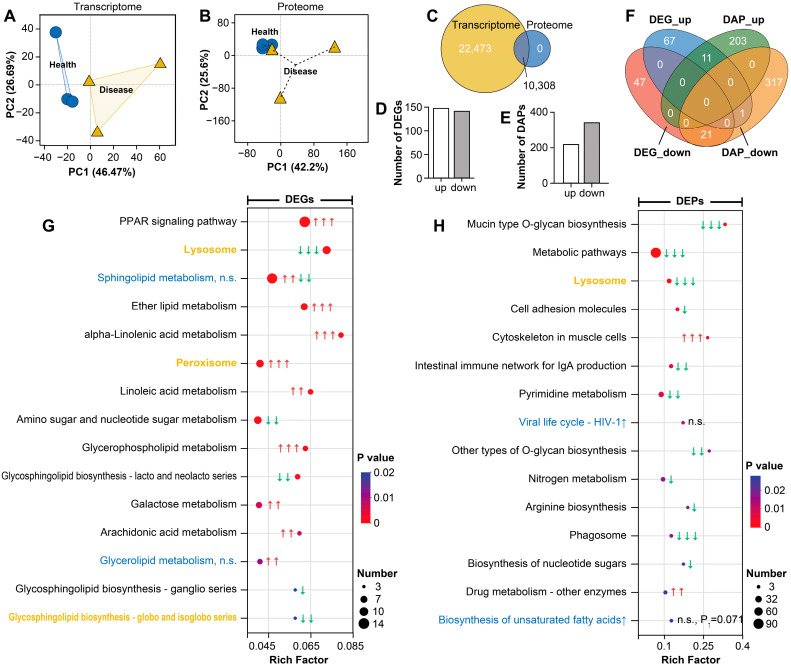
Transcriptomic and proteomic analysis in grass carp intestine during OWS. (**A**) PCA of transcriptomic data. (**B**): PCA of proteomic data. (**C**): Venn diagram of co-annotated genes and proteins. (**D**): Statistics of differentially expressed genes (DEGs) identified using the DESeq2 algorithm with Benjamini–Hochberg (BH) correction. (**E**): Statistics of differentially abundant proteins (DAPs) identified using Welch’s *t*-test. (**F**): Venn diagram of overlapping DEGs and DAPs. (**G**,**H**): KEGG enrichment results of differentially expressed genes (**G**) and proteins (**H**) between groups. Red arrows indicate significantly up-regulated pathways, while green arrows indicate significantly down-regulated pathways. ↑, ↑↑, and ↑↑↑ denote enrichment *p* < 0.05, <0.01, and <0.001, respectively. Yellow highlights indicate pathways that are consistently enriched and similarly regulated at both the gene and protein levels, and blue highlights indicate pathways that are enriched at both levels but with inconsistent regulation. “n.s.” represents not significant.

A comprehensive quality assessment was performed on transcriptomic and data-independent acquisition (DIA) proteomic data. Transcriptomic sequencing data from all samples exhibited high quality: clean reads per sample ranged from approximately 39.46 to 47.37 million, with Q20 and Q30 scores exceeding 99.1% and 96.0%, respectively. GC content (43.24–46.64%) fell within the expected range, and alignment metrics showed high genome mapping rates (unique mapping rates between 83.06% and 90.49%; Table 1). Proteomic data quality was also robust, as indicated by the normal distribution of peptide lengths, supporting the reliability of subsequent quantitative analyses. Principal component analysis (PCA) revealed partial but discernible separation between control and disease groups at both transcriptomic (Figure 2A) and proteomic (Figure 2B) levels, suggesting moderate differences in gene and protein expression profiles. Integrated annotation identified 10,308 co-annotated genes/proteins (Figure 2C). Differential expression analysis revealed 288 significantly differentially expressed genes (DEGs; Figure 2D) and 557 differentially abundant proteins (DAPs; Figure 2E). Among these, 11 molecules were consistently upregulated and 21 were consistently downregulated at both transcript and protein levels (Figure 2F). These coordinated changes suggest that these molecules may be subject to post-transcriptional regulation and could play pivotal roles in the disease process.

At the molecular level, integrated transcriptomic and proteomic profiling revealed coordinated alterations in pathways relevant to cellular degradation, lipid and glycan metabolism, and redox balance. Transcriptome analysis showed predominance of upregulated pathways such as PPAR signaling, ether lipid metabolism, and alpha-linolenic acid metabolism, while pathways related to lysosome-associated functions, amino-sugar and nucleotide-sugar metabolism, and glycosphingolipid biosynthesis were comparatively downregulated (Figure 2G). Proteomic data exhibited a greater number of downregulated pathways, including mucin-type O-glycan biosynthesis, general metabolic pathways, and lysosome-related processes; structural components associated with muscle cell cytoskeleton were among the upregulated categories (Figure 2H). Importantly, several pathways showed concordant changes at both the mRNA and protein levels, most notably lysosome-related functions, peroxisome biology, and glycosphingolipid biosynthesis of the globo and isoglobo series. This cross-layer concordance strengthens confidence in these signals and suggests a coordinated perturbation of intracellular degradation capacity, glycan/lipid processing, and oxidative metabolism in OWS-affected intestines.

### 3.3. Expression of Key Molecules in Consistently Altered Pathways

**Figure 3:**
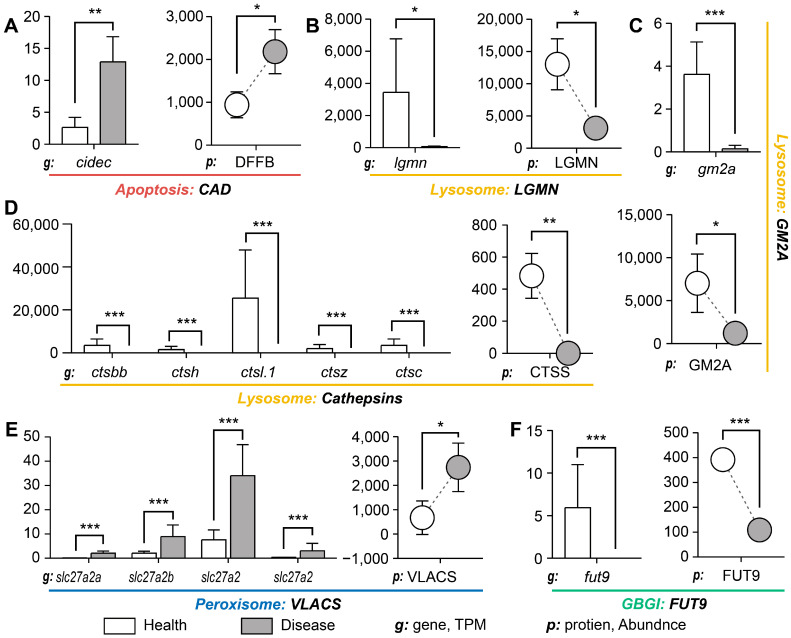
Expression levels of selected genes and proteins in pathways altered during OWS. Panels show relative expression of selected apoptosis-, lysosome-, peroxisome-, and glycosylation-related molecules. Transcriptomic results (*n* = 3 per group) and proteomic results (*n* = 3 per group) are presented as mean ± SD. Statistical significance was determined using the Wilcoxon rank-sum test; *, **, *** indicate *p* < 0.05, <0.01 and <0.001, respectively.

Several individual genes and proteins from pathways that were concordantly altered across omics layers displayed expression patterns consistent with histological injury and barrier compromise. The pro-apoptotic factor CAD was increased in OWS intestines [12] (Figure 3A), consistent with enhanced epithelial apoptosis and exfoliation observed histologically (Figure 1C). Components linked to lysosomal proteolysis showed mixed responses: legumain (LGMN) and several cathepsins were upregulated at the transcript and/or protein level, whereas GM2 activator protein (GM2A) was reduced (Figure 3B–D). The upregulation of proteases such as LGMN and cathepsins may reflect elevated lysosomal proteolytic capacity or a compensatory response to increased substrate load (for example, damaged proteins or microbial debris); conversely, reduced GM2A, a lipid activator involved in ganglioside processing, could impair ganglioside trafficking and membrane lipid homeostasis, with potential consequences for membrane stability and mucus composition [13,14].

Peroxisome-associated changes included upregulation of a very-long-chain acyl-CoA synthetase (VLACS) (Figure 3E), suggesting shifts in fatty acid metabolism that could increase reactive oxygen species (ROS) production and oxidative stress in the epithelium [15]. In parallel, downregulation of FUT9 and related glycosphingolipid biosynthetic enzymes (Figure 3F) indicates altered mucin fucosylation and glycan composition, which may weaken the glycocalyx and affect microbial adhesion [16]. Taken together, these observations are consistent with a model in which altered lysosomal and peroxisomal activity, together with disrupted glycosylation, converge to compromise epithelial integrity and mucosal barrier function. However, these mechanistic links are inferential: functional confirmation (e.g., assays of lysosomal enzyme activities, lysosomal flux/autophagy, targeted lipid/glycan profiling, and measurements of oxidative damage) will be required to establish causality.

### 3.4. OWS Impairs Intestinal Barrier Function and Triggers Intestinal Microbial Dysbiosis

**Figure 4:**
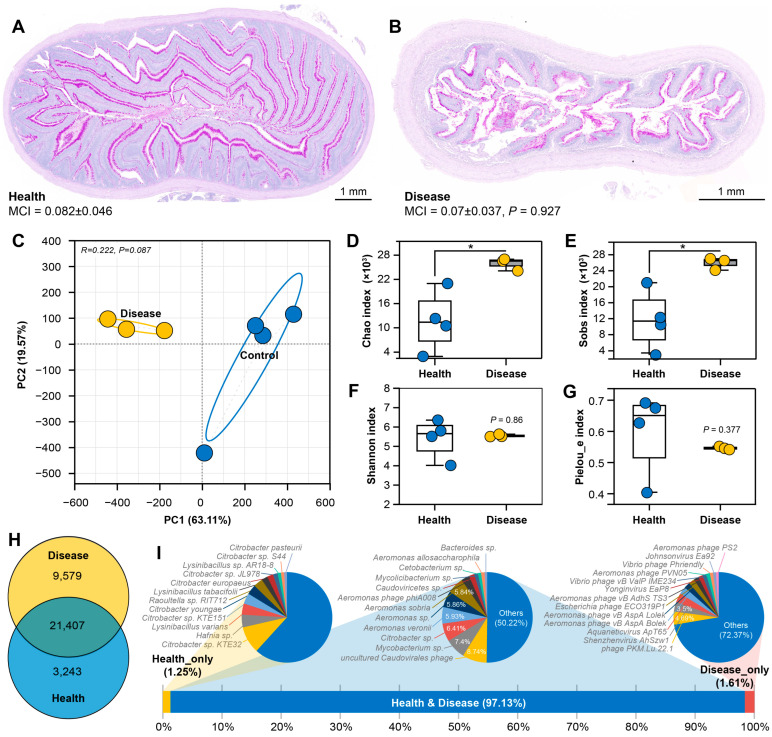
Intestinal mucous cells and basic intestinal microbiota features in grass carp during OWS. (**A**): Intestinal mucous cells in healthy grass carp (AB-PAS staining). (**B**): Intestinal mucous cells in diseased grass carp during OWS (AB-PAS staining); MCI (Mucous Cell Index) was calculated as: MCI = mucous cell area/intestinal section area. (**C**): PCA plot based on metagenomic profiles. (**D**,**E**): Alpha diversity indices (Chao, Sobs) of the intestinal microbiota between groups, reflecting species richness of the microbial community (Wilcoxon rank-sum test, two-tailed); * indicates *p* ≤ 0.05. (**F**): Alpha diversity index (Shannon) of the intestinal microbiota between groups, reflecting species diversity. (**G**): Alpha diversity index (Pielou_e) of the intestinal microbiota between groups, reflecting species evenness. (**H)**: Venn diagram illustrating species composition in intestinal contents between groups based on the NR database. (**I**): Overall composition of taxa unique to or shared between groups based on the NR database.

OWS exposure resulted in compromised intestinal barrier integrity and concomitant microbial dysbiosis, in which aberrant proliferation of viral communities may act as a pivotal contributing factor. Despite histological evidence of mucosal damage, neither mucous cell area nor mucous cell index (MCI) differed significantly between groups (Figure 4A,B). Metagenomic analysis of intestinal contents was conducted to evaluate the impact of OWS on gut microbiota composition. Quality control of the sequencing data indicated robust data quality, with clean reads per sample ranging from 80.37 to 124 million. After optimization and assembly, contig counts varied between 5774 and 405,204 per sample, with the disease group generally yielding higher numbers. Assembly continuity was satisfactory (N50: 535–834 bp; N90: 332–388 bp), and ORF prediction resulted in 19,622 to 660,844 unique genes per sample (Table 2), suggesting substantial inter-sample variation in microbial genetic potential. Principal component analysis (PCA) based on overall microbial community structure revealed clear separation between control and disease groups (Figure 4C), confirming a significant OWS-induced alteration of the gut microbiota. The increased unique gene count in the disease group implies enhanced microbial community complexity and/or the emergence of novel strains or viral sequences. Microbiota analysis demonstrated a marked increase in Chao and Sobs indices, reflecting enhanced species richness, in the OWS-affected group (Figure 4D,E). In contrast, the Shannon and Pielou_e indices, indicative of species diversity and evenness, respectively, showed no significant difference (Figure 4F,G). These shifts suggest a microbial community characterized by elevated colonization density but unchanged dominance evenness among taxa. Taxonomic annotation against the NR database identified 34,229 microbial species across both groups, with 21,407 species shared (Figure 4H). The number of species uniquely annotated in the diseased group far exceeded that in the control group. Species unique to each group constituted a minor fraction of the total CPM (1.25% in controls vs. 1.61% in the diseased group) and were thus non-core components. Uniquely annotated species in controls belonged primarily to bacterial taxa, whereas those in the diseased group were dominated by viruses and bacteriophages. High-abundance shared species included *uncultured Caudovirales phage*, *Mycobacterium* sp., *Citrobacter* sp., and members of the *Aeromonas* genus (Figure 4I).

### 3.5. Intestinal Microbiota Correlation with Host DEGs

**Figure 5:**
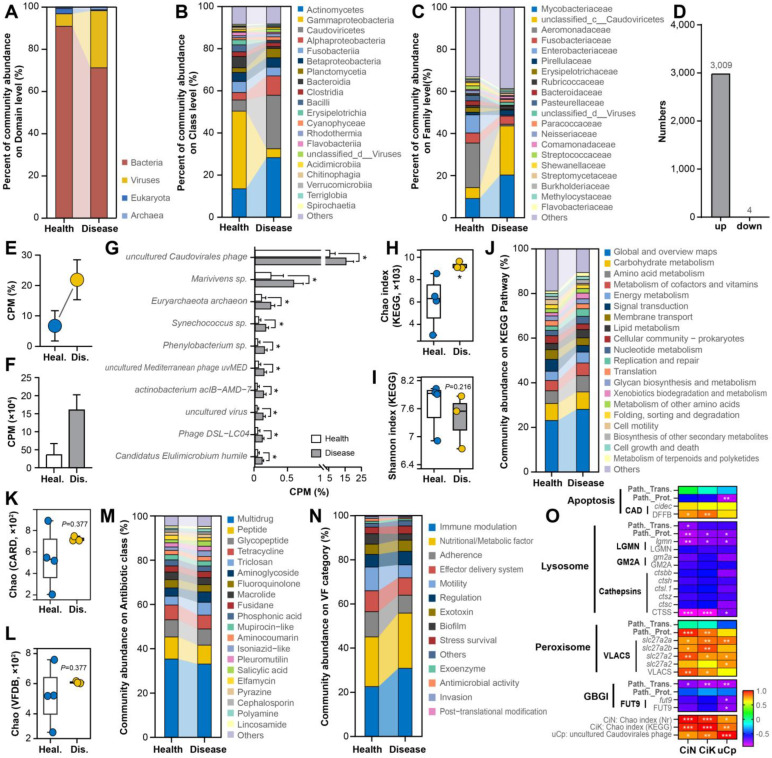
Intestinal microbiota taxonomic abundance, functional contribution, and correlation with host DEGs. (**A**–**C**): Composition of the intestinal microbiota between groups, showing the relative abundance of major microbial taxa at the domain (**A**), class (**B**), and family (**C**) levels. (**D**): Statistics of significantly altered microbial features in the diseased group relative to controls (Shapiro–Wilk normality test and Welch’s *t*-test, two-tailed). (**E**): Proportion of total CPM accounted for by all differentially abundant microbial features. (**F**): Total CPM abundance of all differentially abundant microbial features. (**G**): Expression profiles of the top 10 differentially abundant species by abundance (Welch’s *t*-test, two-tailed); * indicate *p* < 0.05. (**H**,**I**): KEGG-based alpha diversity indices (Chao and Shannon) between groups (Wilcoxon rank-sum test, two-tailed); * indicate *p* < 0.05. (**J**): Composition of microbiota-associated KEGG pathways between groups. (**K**,**L**): Chao alpha diversity indices based on CARD and VFDB databases, respectively (Wilcoxon rank-sum test, two-tailed). (**M**,**N**) Composition of antibiotic resistance genes (**M**) and virulence factors (**N**) harbored by the microbiota between groups. (**O**) Spearman correlation analysis between species abundance, species-associated KEGG pathway changes (Chao index), and the core species *uncultured Caudovirales phage* with target pathways and genes from transcriptomic and proteomic data. ***, **, * represent significance levels of 1%, 5%, and 10%, respectively.

Further compositional profiling revealed a domain-level decline in bacterial and fungal abundance accompanied by a pronounced increase in viral load (Figure 5A), highlighting a potential viral-centric reshaping of the microbial ecosystem under OWS stress. At the class level, while core taxa such as Actinomycetes, Gammaproteobacteria, Caudoviricetes, and Alphaproteobacteria were present in both groups, significant shifts were observed in Gammaproteobacteria and Caudoviricetes, among others (Figure 5B). This shift was refined at the family level to taxa including *unclassified_c__Caudoviricetes* and *Aeromonadaceae* (Figure 5C). Differential statistical testing identified 3013 species that significantly differed between groups, of which 3009 were significantly upregulated in the diseased group (Figure 5D). Although individually low in abundance, the collective CPM of these differential species increased significantly (Figure 5E,F), with their proportion rising from 6.71% in controls to 21.89% in the diseased group (Figure 5E). Core differential species included *uncultured Caudovirales phage*, *Marivivens* sp., and *Euryarchaeota archaeon*, with *uncultured Caudovirales phage* being the most abundant (Figure 5G). KEGG-based alpha diversity analysis indicated a significant increase in the richness of microbiota-associated functions in the diseased group (Figure 5H), but no significant difference in functional diversity (Shannon index; Figure 5I). Compositional analysis of KEGG pathways showed an increase in ‘Global and overview maps’ in the diseased group, while other functional categories generally decreased (Figure 5J), suggesting a potential weakening of commensal metabolic capabilities. Analysis of antibiotic resistance genes (ARGs) and virulence factors (VFs) revealed no significant differences in the richness of these features between groups (Figure 5K,L). The major ARG types (e.g., Multidrug, Peptide, Glycopeptide; Figure 5M) and VF categories (e.g., Immune modulation, Nutritional/Metabolic factor, Adherence; Figure 5N) were similar between groups. Finally, correlation analysis assessed relationships between species richness, functional richness, the core species *uncultured Caudovirales phage*, and key intestinal pathways altered in OWS (Figure 5O). Results showed a strong positive correlation between changes in species richness and functional richness. Furthermore, microbiota changes correlated with alterations in core pathways and associated genes/proteins (e.g., *CAD*, *VLACS*, *CTSS*). Overall, the richness of the microbial community correlated more strongly with intestinal pathway alterations than did the abundance of the single core phage species, underscoring a link between global microbial community restructuring and intestinal pathology during OWS.

## 4. Discussion

The fish intestine constitutes a complex microecosystem shaped by diverse microbial communities, with a collective genome (gut metagenome) forming a functional holobiont with the host that plays indispensable roles in nutrient metabolism, immune development, and pathogen resistance [17]. OWS involves an intestinal ecological breakdown, and diseased fish exhibited concurrent failure of three intestinal barrier defenses: physical, microbial, and immunological. Several differentially expressed molecules form a network linking barrier dysfunction to disrupted host–microbiota crosstalk. Multi-omics highlight lysosomal biology and glycosphingolipid metabolism: upregulated lysosomal proteases (e.g., legumain, cathepsins) with downregulated GM2 activator (GM2A) and glycosyltransferase FUT9 signal coordinated disturbances in degradation and glycan/lipid synthesis. Reduced GM2A likely perturbs ganglioside metabolism and membrane stability [14], and downregulated FUT9 disrupts globo-/isoglobo-series glycosphingolipid biosynthesis, altering glycosylation patterns that determine microbial adhesion and mucoglycolytic barrier integrity [18,19,20,21]. Altered lysosomal function may also impair pathogen handling and antigen presentation, reshape the mucosal immune microenvironment and reinforce dysbiosis [22,23]. Coordinated disruptions in lysosomal and peroxisomal pathways (e.g., VLACS upregulation) may further increase oxidative/lipid stress and weaken tight-junction integrity. Our multi-omics data further demonstrate that microbial dysbiosis in OWS strongly correlates with host transcriptional and proteomic signatures of barrier dysfunction. Specifically, downregulation of lysosome-associated pathways and glycosphingolipid biosynthesis at both RNA and protein levels, and disrupted nucleotide metabolism, suggest impaired epithelial turnover, compromised mucosal defenses, and altered metabolic crosstalk between host and microbiota. Across vertebrate systems, microbiome imbalance, especially at the community level, has been linked to increased epithelial permeability and mucosal inflammation through microbial metabolites and immune modulation [24,25,26].

The strong correlation between microbial richness and host gene expression related to immunity, metabolism, and epithelial integrity supports an ecological model: elevated richness reflects unstable microbiota with reduced functional resilience and greater potential for opportunism, provoking aberrant inflammation and tissue damage. Future work on microbial metabolites, mucosal immune profiling, and barrier function assays will be critical in fish models. Despite limited replicates from natural outbreaks, matched high-depth multi-omics convergence supports our conclusions. Together, these results support a model in which cold stress triggers global gut microbiome restructuring, characterized by increased richness and viral expansion, which is intimately linked to intestinal barrier failure and immune imbalance in OWS, thereby highlighting host–microbe interactions as targets for prevention and intervention.

A key observation in OWS fish was the expansion of viral communities, particularly members of the *Caudoviricetes* class (formerly *Caudovirales* phages). Bacteriophages such as *Caudoviricetes* predominantly infect bacterial hosts and modulate bacterial community dynamics through lytic and lysogenic cycles. Human studies have shown that gut virome dysbiosis, driven by expansion of *Caudovirales* phages, can influence intestinal immunity and barrier function by altering bacterial community structure and triggering host immune responses, for example, via TLR-mediated pathways and subsequent inflammatory signaling that impacts epithelial integrity [27]. Phage-bacterium interactions can reshape bacterial ecological networks, potentially amplify opportunistic species while deplete beneficial taxa, which in turn may perturb host metabolic and immune homeostasis. Such phage-driven community shifts have been implicated in gastrointestinal disorders in mammals, although causal links require further mechanistic studies [28]. In our data, phage increase co-occurred with higher microbial richness and correlated with downregulation of lysosomal genes (e.g., *lgmn*, *ctsl.1*) and lipid transport genes (e.g., *slc27a2* family). Although *Caudoviricetes* are not known to infect fish cells directly [29], phage-driven bacterial lysis and released microbial products can increase lysosomal substrate load and alter host–microbe signaling. Consistent downregulation of lysosome and glycosphingolipid biosynthesis pathways at the transcript and protein levels indicates impaired degradation, altered membrane and glycan biology, and disrupted lipid handling, all of which are processes essential for epithelial maintenance [30,31]. Therefore, the observed viral expansion is not merely a correlative feature but may act as an integral component of the dysbiotic cascade, directly contributing to the disruption of host mucosal homeostasis and barrier function in OWS.

## 5. Conclusions

Integrated multi-omics analyses indicate that OWS involves a cold-induced disruption of intestinal barrier homeostasis rather than a pathogen-driven process. OWS fish show downregulation of lysosome-related and glycosphingolipid biosynthesis pathways, consistent with impaired epithelial maintenance, alongside global gut microbiome restructuring characterized by increased richness and expansion of *Caudoviricetes* bacteriophages. Notably, overall microbial community changes, rather than individual taxa, are most strongly associated with host gene expression linked to immunity and epithelial integrity. Together, these findings support an ecological model in which cold stress promotes microbiome instability and barrier dysfunction, increasing susceptibility to intestinal injury during OWS.

## Figures and Tables

**Table 1:** Transcriptomic sequencing quality metrics.

Groups	Raw Bases	Raw Reads	Clean Reads	Q20 (%)	Q30 (%)	GC Content (%)	Total Mapped	Multiple Mapped	Uniquely Mapped
Health-1	6,656,462,936	44,082,536	43,755,852	99.19	96.24	45.02	41,217,290 (94.2%)	2,618,981 (5.99%)	38,598,309 (88.21%)
Health-2	6,007,891,964	39,787,364	39,466,050	99.11	96.02	43.86	37,408,882 (94.79%)	1,879,531 (4.76%)	35,529,351 (90.03%)
Health-3	7,198,904,766	47,674,866	47,370,268	99.24	96.34	45.34	44,864,234 (94.71%)	2,639,734 (5.57%)	42,224,500 (89.14%)
Disease-1	6,656,812,652	44,084,852	43,790,264	99.18	96.05	46.64	41,856,328 (95.58%)	2,228,418 (5.09%)	39,627,910 (90.49%)
Disease-2	6,537,322,124	43,293,524	43,059,992	99.3	96.42	46.28	42,011,024 (97.56%)	3,520,185 (8.18%)	38,490,839 (89.39%)
Disease-3	6,529,803,230	43,243,730	42,889,582	99.12	96.02	43.24	39,580,005 (92.28%)	3,957,316 (9.23%)	35,622,689 (83.06%)

**Table 2:** Metagenomic sequencing and assembly statistics.

Samples	Insert Size (bp)	Read Length (bp)	Raw Reads	Raw Base (bp)	Clean Reads	Optimized Reads	Contigs	N50 (bp)	N90 (bp)	ORFs	Unique Number
Control-1	428	150	124,391,100	18,783,056,100	123,186,102	457,848	5774	834	388	3672	19,622
Control-2	457	150	102,378,636	15,459,174,036	101,370,020	1,112,266	17,170	593	346	17,303	96,743
Control-3	438	150	123,303,518	18,618,831,218	121,939,648	1,378,324	18,704	535	335	18,762	111,748
Disease-1	446	150	82,140,074	12,403,151,174	80,372,894	28,444,206	336,213	556	332	442,610	631,620
Disease-2	476	150	91,522,452	13,819,890,252	90,327,434	73,085,184	405,204	585	333	557,087	660,844
Disease-3	432	150	91,985,638	13,889,831,338	90,332,036	11,505,294	160,110	570	334	207,368	477,914

## Data Availability

The authors confirm that the data supporting the findings of this study are available within the article. Raw sequencing data have been deposited in the China National Center for Bioinformation (CNCB). Metagenomic data are available under GSA accession number CRA033205, transcriptomic data under accession number CRA033192, and proteomic data under accession number OMIX012914.
